# Prenatal Phthalate Exposure Is Associated with Childhood Behavior and Executive Functioning

**DOI:** 10.1289/ehp.0901470

**Published:** 2010-01-28

**Authors:** Stephanie M. Engel, Amir Miodovnik, Richard L. Canfield, Chenbo Zhu, Manori J. Silva, Antonia M. Calafat, Mary S. Wolff

**Affiliations:** 1 Department of Preventive Medicine, Mount Sinai School of Medicine, New York, New York, USA; 2 Division of Nutritional Sciences, College of Human Ecology, Cornell University, Ithaca, New York, USA; 3 National Center for Environmental Health, Centers for Disease Control and Prevention, Atlanta, Georgia, USA

**Keywords:** attention deficit hyperactivity disorder, BASC, BRIEF, environmental exposure, phthalate

## Abstract

**Background:**

Experimental and observational studies have reported biological consequences of phthalate exposure relevant to neurodevelopment.

**Objective:**

Our goal was to examine the association of prenatal phthalate exposure with behavior and executive functioning at 4–9 years of age.

**Methods:**

The Mount Sinai Children’s Environmental Health Study enrolled a multiethnic prenatal population in New York City between 1998 and 2002 (*n* = 404). Third-trimester maternal urines were collected and analyzed for phthalate metabolites. Children (*n* = 188, *n* = 365 visits) were assessed for cognitive and behavioral development between the ages of 4 and 9 years.

**Results:**

In multivariate adjusted models, increased log_e_ concentrations of low molecular weight (LMW) phthalate metabolites were associated with poorer scores on the aggression [β = 1.24; 95% confidence interval (CI), 0.15– 2.34], conduct problems (β = 2.40; 95% CI, 1.34–3.46), attention problems (β = 1.29; 95% CI, 0.16– 2.41), and depression (β = 1.18; 95% CI, 0.11–2.24) clinical scales; and externalizing problems (β = 1.75; 95% CI, 0.61–2.88) and behavioral symptom index (β = 1.55; 95% CI, 0.39–2.71) composite scales. Increased log_e_ concentrations of LMW phthalates were also associated with poorer scores on the global executive composite index (β = 1.23; 95% CI, 0.09–2.36) and the emotional control scale (β = 1.33; 95% CI, 0.18– 2.49).

**Conclusion:**

Behavioral domains adversely associated with prenatal exposure to LMW phthalates in our study are commonly found to be affected in children clinically diagnosed with conduct or attention deficit hyperactivity disorders.

Biomonitoring of phthalate metabolites has identified virtually ubiquitous human exposure internationally and for all age groups (Centers for Disease Control and Prevention 2005). Phthalate metabolites have been detected in many body tissues including urine, blood, semen, amniotic fluid, and breast milk (National Research Council 2008). Despite the relatively rapid clearance of phthalate metabolites, urine phthalate biomarker measurements are relatively stable over periods of days to months (Adibi et al. 2008; Hauser et al. 2004; Teitelbaum et al. 2008), probably because exposure sources and patterns of usage of phthalate-containing products are common and fairly consistent (Hauser et al. 2004; Teitelbaum et al. 2008).

At least 10 different phthalates are used commercially as plasticizers, solvents, antifoam agents, or alcohol denaturants. High-molecular-weight phthalates (HMWP) [e.g., di(2-ethylhexyl) phthalate (DEHP)] can be found in tubing, vinyl flooring, and wall covering. Low-molecular-weight phthalates (LMWP) (e.g., diethyl phthalate) more commonly can be present in personal care products (fragrances, shampoo, cosmetics, and nail polish). Phthalates are also found as both inert and active ingredients in some pesticide formulations. Human exposure to phthalates can occur through inhalation, ingestion, and dermal contact. Once absorbed, they are rapidly metabolized to monoesters, and the high-molecular-weight monoesters can undergo further oxidation to form oxidative metabolites (National Research Council 2008).

Some phthalates have been regulated in consumer products in Europe and the United States (California Safe Cosmetics Act 2005; Consumer Product Safety Improvement Act 2008; Directive 2005/84/EC of the European Parliament and of the Council 2005). The regulations in the United States are aimed at reducing childhood exposure to DEHP, dibutyl phthalate (DBP), and benzyl butyl phthalate by regulating their presence in bath toys or other small plastic toys that can be placed in the mouth easily. However, in some circumstances, these phthalates have been replaced with others (e.g., di-isononyl- or di-isodecyl-phthalates for DEHP) that remain unregulated. Regulatory action was based mainly on studies of male reproductive toxicity in both animals and humans, which is related to testicular-based androgen insufficiency (National Research Council 2008).

Antagonistic effects of phthalates on the thyroid gland *in vivo* and thyroid tissue *in vitro* have been reported (Hinton et al. 1986; Pereira et al. 2007; Poon et al. 1997; Price et al. 1988; Sugiyama et al. 2005). DBP has been associated with a dose-dependent decrease in circulating triiodothyronine and thyroxine (T_4_) in rats (O’Connor et al. 2002). In humans, low serum free T_4_ was associated with high urinary concentrations of monobutyl phthalate (a metabolite of DBP) (Huang et al. 2007) and of mono(2-ethylhexyl) phthalate (a metabolite of DEHP) during pregnancy (Meeker et al. 2007). Recently, phthalate exposure in childhood was associated with attention deficit hyperactivity disorder (ADHD) in a cross-sectional study of Korean school children between the ages of 8 and 11 years (Kim et al. 2009).

## Objective

The objective of the Mount Sinai Children’s Environmental Health Center is to investigate the role of prenatal toxicant exposures on childhood growth and neurodevelopment. We recently reported a relationship between prenatal maternal concentrations of phthalate metabolites and altered neonatal behavior, specifically in the orientation and motor scales and their overall quality of alertness, examined within 5 days of delivery (Engel et al. 2009). The consequences of prenatal exposure on neurobehavioral development during childhood have not previously been reported. Therefore, we examined these relationships in a subset of our cohort who returned for follow-up visits between 4 and 9 years of age.

## Methods

### Enrollment of birth cohort and child follow-up

The Mount Sinai Children’s Environmental Health study enrolled a prospective multiethnic cohort of primiparous women with singleton pregnancies. Women presented for prenatal care either at the Mount Sinai Diagnostic and Treatment Center, which serves the predominantly minority East Harlem population, or at one of two private practices on the Upper East Side of Manhattan. Four hundred seventy-nine mother–infant pairs were successfully recruited. Women delivered at the Mount Sinai Medical Center between May 1998 and July 2001 (Berkowitz et al. 2003, 2004). Seventy-five women were excluded for reasons detailed elsewhere (Engel et al. 2007), including extreme prematurity; the final cohort was 404 women for whom birth data were available. Questionnaires were administered to participants during the third trimester of pregnancy to obtain information on sociodemographic characteristics, medical history, and lifestyle factors. A maternal spot urine sample was obtained between 25 and 40 weeks’ gestation (mean, 31.2 weeks). Delivery characteristics and birth outcomes were obtained from a perinatal database maintained within the Mount Sinai Department of Obstetrics, Gynecology and Reproductive Science.

Women were invited to return for three follow-up visits when their children were between 4 and 9 years of age. We attempted to complete interviews in each of these three periods: 4.5–5.5 years; 6–6.5 years; and 7–9 years. The number of visits per child ranged from one to three (approximately 40% came once, 26% came twice, 34% came three times), totaling 365 visits completed by 188 children (Table 1). Among these were five children who were not included in the original birth cohort analysis (*n* = 404) because they were not delivered at Mount Sinai (and therefore birth outcome information was unavailable), although they returned for follow-up study visits. The study was approved by the Institutional Review Board of the Mount Sinai School of Medicine; participants provided written informed consent before the study, and children ≥ 7 years of age provided assent.

### Phthalate metabolite measurements

Sufficient maternal urine remained for 177 women to measure phthalate metabolite concentrations. Maternal urine samples were analyzed at the Centers for Disease Control and Prevention for 10 phthalate metabolites. Methods and quality control procedures have been described previously (Kato et al. 2005; Silva et al. 2008). To limit the number of statistical tests performed, phthalate metabolites were grouped into two categories defined by the molecular weight of the monoesters [high (> 250 Da) and low (< 250 Da)], as they have similar biologic activity as their parent diesters and come from similar environmental sources (Wolff et al. 2008). The concentrations, by molar sum and individual metabolite, have been previously reported (Wolff et al. 2008). To account for urine dilution, we included log-creatinine in models where metabolites were considered continuous, log-transformed variables. When metabolites were considered in tertiles, creatinine-based concentrations (micromoles per gram creatinine) were used to derive tertile cut points.

### Behavior and executive functioning outcomes

At each visit, mothers completed the parent-report forms of the Behavior Rating Inventory of Executive Function (BRIEF), and the Behavior Assessment System for Children-Parent Rating Scales (BASC-PRS). These are standardized instruments commonly used in both research and clinical environments that have been validated in diverse samples and have known psychometric properties (Gioia et al. 2000; Reynolds and Kamphaus 1998). Mothers also provided current sociodemographic and other information on their children. Questionnaires were available in both Spanish and English.

The BRIEF is an 86-item questionnaire designed to assess executive cognitive function in children 5–18 years of age. Executive functions are used to achieve goals that require planning and holding in memory a multistep sequence of thoughts or actions, to monitor and control attention and emotion, to inhibit inappropriate behaviors, and to formulate mental models based on life experiences (Pennington et al. 1997). The BRIEF is composed of eight clinical scales:

- Inhibit—the ability to control impulses
- Shift—the ability to transition between situations
- Emotional control—the ability to modulate emotional responses
- Initiate—the ability to begin a task
- Working memory—the ability to retain information for task completion
- Plan/organize—the ability to anticipate future events, set goals, and develop a systematic plan of action
- Organization of materials—the ability keep workspace orderly
- Monitor—the ability to asses personal performance and to register the effect of one’s own behavior on others.

These eight scales generate two broad indexes: Behavioral Regulation Index (BRI) and Metacognition Index (MI). An overall score, the Global Executive Composite (GEC), is obtained from the raw scores for the MI and BRI. For all scales, higher scores indicate worse executive functioning.

The BASC (Reynolds and Kamphaus 1998) is designed to evaluate problematic behaviors in children and adolescents 2.5–18 years of age. The BASC-PRS includes nine clinical scales to assess a child’s adaptive and problem behaviors in home and community settings. Parents respond to 130 items on a 4-point scale that ranges from never to almost always. Externalizing problems is a composite scale derived from the hyperactivity (including both hyperactivity and impulsivity items), aggression, and conduct problems scale items. Internalizing problems is a composite of the anxiety, depression, and somatization scale items. Three additional scales are attention problems, atypicality, and withdrawal. The adaptive behavior skills composite combines information from the adaptability, social skills, and leadership scale items. The Behavioral Symptoms Index (BSI) is the apical summary score that assesses the overall level of behavioral functioning. For the clinical and composite scales, higher scores indicate more problem behaviors. For the adaptive scales, lower scores indicate more problem behaviors. Thresholds delineating an at-risk or clinically significant scaled score have been established. For the clinical scales, the at-risk threshold is a *T*-score of 60–69, and the clinically significant threshold is a *T*-score of ≥ 70. For the adaptive scales, the at-risk threshold is a *T*-score of 31–40, and the clinically significant threshold is a *T*-score of ≤ 30.

The BRIEF and BASC are standardized for age and sex and provide *T*-scores with a mean (± SD) of 50 ± 10. Both instruments have good internal consistency, test–retest reliability, content and construct validity, and convergent discriminate validity (Pizzitola 2002; Reynolds and Kamphaus 1998).

### Statistical analyses

We analyzed data using SAS version 9.2 (SAS Institute Inc., Cary, NC). Both the BASC and BRIEF include standard validity scales to identify problematic observations. For the BASC, the Infrequency Index (F) includes items that are rarely endorsed when the assessment is valid and can indicate an excessively negative evaluation of the child, a failure to follow instructions, random responding, or difficulty reading. Raw *F*-scores on the BASC Parent Rating Scales (PRS) can vary from 0 to 6, with higher scores warranting closer review. All surveys with *F*-scores greater than 3 (*n* = 2 surveys) were excluded *a priori,* because the validity of these surveys is questionable. In the case of *F*-scores of 2 (*n* = 15 surveys) or 3 (*n* = 10 surveys), a committee of three investigators reviewed the child’s records for parental reports of clinically diagnosed or treated psychiatric or neurologic problems and current or past usage of psychotropic medications. The reviewers also looked for evidence of invalid, inconsistent, or unusual response patterns. After review, we excluded 12 surveys for the following reasons: language difficulty (*n* = 2), evidence of random responding (*n* = 7), and overly negative or unrealistic evaluation of child’s behavior (*n* = 3). We did not exclude surveys with *F*-scores of 2 or 3 if the parent reported negative behaviors and the child was currently receiving treatment for or had previously been diagnosed with psychiatric or neurologic problems.

**Figure 1:**
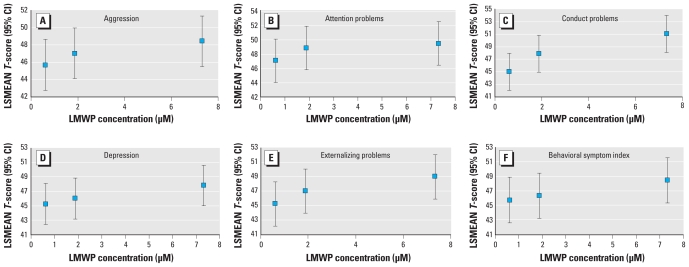
Adjusted mean BASC T-score and 95% CIs for aggression (*A*), attention problems (*B*), conduct problems (*C*), depression (*D*), externalizing problems (*E*), and BSI (*F*), plotted at the median creatinine-based phthalate biomarker concentration by tertile of exposure to assess linearity in dose response. LSMEAN, least-square mean. Means were adjusted for race, sex, educational level of the primary caretaker, and marital status of the primary caretaker. All affected domains appeared to be monotonically associated with phthalate biomarker level, with the exception of attention problems, which appeared to plateau at 2 μM.

Children were invited to return for three visits, but compliance varied. For children who returned for more than one visit, we took the average of scale-specific *T*-scores. Because *T*-scores are age-standardized, no additional adjustment for child age in the model was included. We conducted multivariate analyses using PROC GLM (SAS Institute Inc.). Independent variables were chosen based on their relationships with the outcomes of interest, as well as their correlation with phthalate metabolite concentrations. Given that phthalates are reported to be reproductive toxicants and exhibit hormonal antagonism, we *a priori* hypothesized that there may be sex interactions. Therefore, we first examined the significance of sex–phthalate interactions (α = 0.10), before considering sex as a possible confounder. We used a backward elimination method to obtain the most parsimonious model, eliminating covariates that did not change the estimate of the main effect by at least 10%. All models were adjusted for urinary creatinine concentration. As a first step, effects of phthalate metabolite concentration were examined using creatinine-based tertiles. Because the trend in the least-square means across the tertiles supported a linear effect, the final models were based on the log_e_-linear term of phthalate metabolite concentrations (Figure 1). Alternative analytic approaches (e.g., repeated measures analyses) were examined to evaluate the consistency of our findings. In these models, the coefficient for visit did not significantly differ from zero, suggesting that scores did not systematically vary across visits. Additionally, the phthalate effects were very similar comparing the average *T*-scores to the repeated measures models; thus, our final models were based on the average score.

## Results

There were slight differences in the characteristics of women who returned for follow-up versus the original birth cohort (Table 1). Compared with the original birth cohort, the women who returned for follow-up tended to be those who were slightly older at the time of enrollment. However, the racial/ethnic mix, educational attainment, marital status, and median urinary concentrations of the metabolites of low and high molecular phthalates were similar. Among the children followed up, 54% were boys, 75% of the children spoke only English at home, and 83% of mothers were the child’s primary caretaker. Those with other caretakers generally reported either father or grandmother.

HMWP were not associated with most of the BASC or BRIEF domains, except that increased log-HMWP was associated with poorer scores on the adaptability scale of the BASC [β = −1.33; 95% confidence interval (CI), −2.53 to −0.14].

Low molecular weight phthalate (LMWP) concentrations were strongly related to a number of clinical and composite scales (Table 2). In multivariate adjusted models, each log-unit increase in LMWP metabolite concentrations was associated with a 1.24-point increase on the aggression scale [95% confidence interval (CI), 0.15–2.34], a 1.29-point increase on the attention problems scale (95% CI, 0.16–2.41), a 2.4-point increase on the conduct problems scale (95% CI, 1.34–3.46), and a 1.18-point increase on the depression scale (95% CI, 0.11–2.24). Although the associations between urinary concentrations of phthalate metabolites and the adaptive scales did not reach conventional levels of statistical significance, there was a consistent pattern of poorer adaptive profiles with increasing metabolite concentrations. This pattern was also evident in the composite Adaptive Skills Index (β = −0.98; 95% CI, −2.05 to 0.09). However, the associations were stronger between phthalate metabolite concentrations and externalizing problems (β = 1.75; 95% CI, 0.61–2.88) and the BSI (β = 1.55; 95% CI, 0.39–2.71). These effects were not modified by the child’s sex. Using creatinine-corrected tertiles of phthalate exposure, we plotted the tertile-specific adjusted mean *T*-score at the median micromolar concentration of metabolites of LMWP to examine the shape of the dose–response relationship (Figure 1). Consistent with the log-linear models, the strongest linear trends were demonstrated for conduct problems and externalizing problems; however, all scales demonstrated monotonically increasing LSMEANS, with the exception of the attention scale, which seemed to suggest a threshold effect at approximately 2 μM.

Even restricting our analyses to BASC surveys with *F*-scores < 2 (*n* = 161), most associations between phthalate exposure and behavior remain among boys. Significant sex–phthalate interactions (i.e., significant differences in the slope of the association between log-LMWP and behavior between girls and boys) were found for the aggression, conduct problems, hyperactivity, externalizing problems, and BSI. There were few significant associations among the girls; however, among boys, increasing log-LMWP metabolite concentrations were associated with poorer *T*-scores for aggression (β = 1.46; 95% CI, 0.17–2.76), attention problems (β = 1.55; 95% CI, 0.22–2.88), and conduct problems (β = 2.79; 95% CI, 1.55–4.03), which was also significant in girls but with a smaller effect magnitude and less precision, externalizing problems (β = 2.08; 95% CI, 0.74–3.42), and for the BSI (β = 1.60; 95% CI, 0.23–2.97) (Table 3).

Few surveys met the threshold scores for at risk or clinically significant (*T*-scores ≥ 60 for the clinical and composite scales or ≤ 40 for the adaptive scales) in this population. However, in a sensitivity analysis, we examined whether greater than the median metabolite concentration was associated with increased risk of scoring in the at-risk or clinically significant range on the scales identified in Table 2. In general, we found that the effects were in the same direction, although only attention problems (*n* = 27 at-risk or clinically significant surveys) reached statistical significance (relative risk = 2.66; 95% CI, 1.06–6.66).

We also examined the metabolite-specific effects on the BASC domains (Table 4). In general, we found consistency between the LMWP sum effects and the metabolite-specific models.

Except for aggression and conduct problems, the LMWP molar sum significance was replicated in at least two of the individual metabolite models for that scale. Although for monobutyl phthalate (MBP) only aggression and externalizing problems were statistically significant, the magnitude of the MBP associations were very similar to the LMWP sum effects for attention problems, adaptability, and the BSI.

There were fewer consistent associations between LMWP metabolite concentrations and executive functioning as measured with the BRIEF. However, similar to what was observed for the BASC, increased log_e_-LMWP metabolite concentrations was associated with poorer scores on the emotional control scale (β = 1.33; 95% CI, 0.18–2.49) and on the GEC index (β = 1.23; 95% CI, 0.09, 2.36) (Table 5). There were no interactions between phthalate exposure and the child’s sex for the BRIEF scales. We also compared the metabolite-specific models with the total LMWP molar sum (Table 6). There were a number of significant associations for monomethyl phthalate (MMP), however, this metabolite has the lowest concentration among all metabolites measured. MBP was associated with poorer scores on working memory (β = 1.53; 95% CI, −0.01 to 3.07), but in general, the molar sum effects were consistent in direction and magnitude with the metabolite specific effects.

## Discussion

We report an association between prenatal exposure to LMWP and poorer parent-rated behavioral and executive functioning profiles for children between the ages of 4 and 9 years. Specifically, higher LMWP metabolite concentrations were associated with poorer scores on the aggression, attention problems, conduct problems, depression, and externalizing problems scales, and for the overall BSI on the BASC. Similarly, poorer executive functioning was indicated by elevated scores on the emotional control scale and on the GEC index of the BRIEF. These effects remained statistically significant among boys, even after restricting the eligible surveys to those with *F*-scores of 0 or 1. All of these effects were consistent with a dose–response gradient.

In psychometric validity studies comparing the BRIEF and BASC (Gioia et al. 2000), the BRIEF emotional control scale is strongly correlated (*r* > 0.6; *p* < 0.01) with the BASC-PRS aggression, anxiety, and depression scales (Gioia et al. 2000). Also in these validity studies, the GEC index is strongly correlated with the BASC-PRS aggression and attention problems scales. We find comparable associations among these measures, supporting the validity of our application of these instruments.

Taken as a whole, the profile of parent-reported behaviors we find associated with prenatal LMWP metabolite concentrations is suggestive of the behavior profiles of children clinically diagnosed with disruptive behavior disorders, for example, oppositional defiant disorder, conduct disorder, or ADHD (Loeber et al. 2009; Reynolds and Kamphaus 1998, Table 13.25) The strongest associations we find are for conduct problems, externalizing problems, and the BSI, and ratings on these parent-report scales typically serve as one element in a clinical diagnosis. The strong associations with the composite scales (externalizing and BSI) reflect the fact that on every scale for which a higher score reflects more problem behaviors, the direction of the association suggests an adverse effect of prenatal LMWP exposure. The association for the GEC on the BRIEF also reflects the consistency in the direction of association on that instrument.

ADHD and disruptive behavior disorders are highly comorbid conditions, and no clinical diagnosis can be based solely on screening instruments reliant on the parent’s perceptions and recollections of their child’s behaviors. The instruments we used include specific items and patterns of responding to identify some types of parent reporting bias (e.g., excessive negativity), but other biases might go undetected. Parents’ perceptions are also based on behaviors occurring in family situations, whereas the child might show different behaviors at school, and parents might be unaware of the child’s subjective experiences of anxiety and other forms of internalization. Although few children in this study met the standard at risk or clinically significant criteria on the BASC-PRS, the patterns across scales and the consistency in findings across instruments warrant additional study on the role of prenatal exposure to LMWP in the emergence of disruptive behavior problems in children.

The mechanism underlying a possible association between phthalates and neurodevelopment has not been established, but may include prenatal disruption of the maternal thyroid hormone system (Breous et al. 2005; Hinton et al. 1986; Huang et al. 2007; Meeker et al. 2007; O’Connor et al. 2002; Pereira et al. 2007; Poon et al. 1997; Price et al. 1988; Sugiyama et al. 2005; Vermiglio et al. 2004) or activation of peroxisome proliferator-activated receptors (Braissant and Wahli 1998; Corton and Lapinskas 2005; Latini et al. 2008; Peters et al. 2000) (Xu et al. 2007). Early pregnancy mild hypothyroxinemia (normal thyroid-stimulating hormone, abnormal free T_4_) was found to be associated with abnormal motor and socialization quotients in children at 18 months of age, which was preventable with early iodine supplementation (Berbel et al. 2009). Additionally, prenatal iodine-deficient hypothyroxinemia has been associated with ADHD in childhood (Vermiglio et al. 2004). However, additional research is needed to investigate these mechanisms and determine their applicability to fetal and child neurodevelopment. We previously reported a relationship between prenatal urinary concentrations of some phthalate metabolites and neonatal behavior as measured by the Brazelton Neonatal Behavioral Assessment Scale (Engel et al. 2009). Here we extend those findings to behavior and executive functioning in childhood.

Direct child exposure to phthalates in the postnatal period may be independently associated with behavioral symptoms or may act cumulatively with prenatal exposure to increase risk. Recently, phthalate exposure during childhood was associated with ADHD in a cross-sectional study of Korean children between the ages of 8 and 11 years (Kim et al. 2009). However, no previous study has examined the influence of prenatal exposure.

Although substantial attrition occurred during the 10 years since this cohort was recruited, we do not believe selection bias can account for our findings. For loss to follow-up to represent a bias in our study, phthalates would have to have a different relationship with behavior and executive functioning in the lost population. We carefully examined the characteristics of the full cohort (*n* = 404) and the follow-up cohort (*n* = 188) and found that the populations were quite similar, except for maternal age at enrollment (Table 1). This was expected, given that the youngest mothers tended to be more difficult to track using the contact information they provided and were also somewhat more likely to move out of the area shortly after delivery. However, there were no differences with respect to phthalate metabolite concentrations. Moreover, none of the mothers in our study were provided with their prenatal phthalate metabolite concentrations; therefore, their evaluation of their child’s behavior was not influenced by their own prenatal exposure level.

Exposure to phthalates is ubiquitous, and although phthalate biological activity may be less severe than that of other endocrine disruptors, the potential public health impact of exposure to phthalates might be greater. We report a pattern of associations between prenatal exposure to some LMWP and childhood behavior and executive functioning that is consistent with domains typically affected in childhood oppositional defiant disorder, conduct disorder, and ADHD. Additionally, most associations were consistent with a monotonic dose response. The reported relations between phthalates and thyroid hormone make such findings biologically plausible; however, additional research is urgently needed to replicate these findings and determine the underlying etiologic mechanism. Preventive measures to reduce exposure during pregnancy may be warranted should these findings be verified.

## Figures and Tables

**Table 1:** Comparison of children followed up with original birth cohort population, Mount Sinai Medical Center, New York, New York, USA, 1998–2008.

	Original birth cohort (*n* = 404)		Children followed up (*n* = 188)^a^		
Population characteristics	*n*	Percent	*n*	Percent	*p*-Value
Maternal age at enrollment (years)					0.04
< 20	142	35.2	58	30.9	
20–24	132	32.7	62	33.0	
25–29	44	10.9	29	15.4	
≥ 30	86	21.3	39	20.7	
Maternal race/ethnicity					0.92
White	86	21.3	38	20.2	
Black	112	27.7	55	29.3	
Hispanic	200	49.5	92	48.9	
Other	6	1.5	3	1.6	
Maternal education					0.09
< High school	118	29.2	46	24.5	
High school	83	20.5	40	21.3	
Some college	103	25.5	57	30.3	
≥ College degree	100	24.8	45	24.0	
Marital status at enrollment					0.10
Married	117	29.0	47	25.0	
Living with father of child	98	24.3	42	22.3	
Single/divorced/widowed	189	46.8	99	52.7	
Smoke during pregnancy (ever)	67	16.6	31	16.9	0.86
Alcohol during pregnancy (ever)	59	14.9	28	15.6	0.78
LMWP (μM/L) (*n* = 177)	2.24^b^	0.90–5.65^c^	1.88^b^	0.83–4.59^c^	0.15
HMWP (μM/L) (*n* = 177)	0.43^b^	0.22–0.91^c^	0.43^b^	0.18–0.90^c^	0.54
Mother is primary caretaker			151	83.0	
Breast-feeding of child (months)					
< 1			70	40.0	
1–4			38	21.7	
> 4			67	38.3	
Retention at each interview period (years)					
4.5–5.5			102	54.3	
6–6.5			113	60.1	
7–9			146	77.7	

a Includes five children who were not included in the birth cohort analyses because they were not born at Mount Sinai.

b Median.

c Interquartile range.

**Table 2:** Prenatal metabolite concentrations of LMWP and the BASC in a multiethnic birth cohort, Mount Sinai Medical Center, New York, New York, USA, 1998–2008.^a^

BASC domain	β per log_e_-unit increase in LMWP (95% CI)
Clinical scales	
Aggression	1.24 (0.15 to 2.34)
Anxiety	0.78 (−0.46 to 2.02)
Attention problems	1.29 (0.16 to 2.41)
Atypicality	0.95 (−0.03 to 1.92)
Conduct problems	2.40 (1.34 to 3.46)
Depression	1.18 (0.11 to 2.24)
Hyperactivity	1.03 (−0.21 to 2.28)
Somatization	0.36 (−0.85 to 1.56)
Withdrawal	0.46 (−0.66 to 1.58)
Adaptive scales	
Adaptability	−1.08 (−2.14 to −0.02)
Leadership	−0.88 (−2.04 to 0.28)
Social skills	−1.04 (−2.14 to 0.05)
Composite scales	
Externalizing problems	1.75 (0.61 to 2.88)
Internalizing problems	0.99 (−0.22 to 2.20)
Adaptive skills	−0.98 (−2.05 to 0.09)
BSI	1.55 (0.39 to 2.71)

a Includes subjects with *F*-scores of 0–3 and adjusted for race, sex, educational level of the primary caretaker, marital status of the primary caretaker, and urinary creatinine. All scales except conduct problems and leadership included 171 subjects. Conduct problems and leadership items are queried only on the 6- to 11-year BASC-PRS, so the number of subjects with these scales was 159.

**Table 3:** Prenatal metabolite concentrations of LMWP and the BASC among boys in a multiethnic birth cohort, Mount Sinai Medical Center, New York, New York, USA, 1998–2008.^a^

BASC domain	β per LMWP log_e_-unit increase in boys (95% CI)	β per LMWP log_e_-unit increase in girls (95% CI)	*p*-Value for interaction
Clinical scales			
Aggression	1.46 (0.17 to 2.76)	0.86 (−0.80 to 2.52)	0.02
Anxiety	0.73 (−0.73 to 2.19)	0.88 (−1.00 to 2.75)	0.42
Attention problems	1.55 (0.22 to 2.88)	0.83 (−0.87 to 2.54)	0.16
Atypicality	0.36 (−0.78 to 1.50)	1.96 (0.49 to 3.42)	0.89
Conduct problems	2.79 (1.55 to 4.03)	1.70 (0.09 to 3.31)	0.02
Depression	1.20 (−0.06 to 2.46)	1.14 (−0.47 to 2.76)	0.19
Hyperactivity	1.39 (−0.08 to 2.85)	0.42 (−1.46 to 2.30)	0.04
Somatization	0.58 (−0.84 to 2.00)	−0.03 (−1.86 to 1.79)	0.39
Withdrawal	0.34 (−0.98 to 1.65)	0.67 (−1.02 to 2.36)	0.85
Adaptive scales			
Adaptability	−1.43 (−2.68 to −0.19)	−0.48 (−2.07 to 1.12)	0.24
Leadership	−0.84 (−2.20 to 0.52)	−0.95 (−2.72 to 0.82)	0.54
Social skills	−1.65 (−2.93 to −0.37)	0.00 (−1.64 to 1.65)	0.14
Composite scales			
Externalizing problems	2.08 (0.74 to 3.42)	1.17 (−0.55 to 2.89)	0.01
Internalizing problems	1.07 (−0.36 to 2.50)	0.86 (−0.98 to 2.69)	0.21
Adaptive skills	−1.19 (−2.45 to 0.08)	−0.63 (−2.25 to 0.98)	0.12
BSI	1.60 (0.23 to 2.97)	1.46 (−0.30 to 3.21)	0.05

a Includes subjects with *F*-scores of 0–1 and adjusted for race, educational level of the primary caretaker, marital status of the primary caretaker, urinary creatinine, and including sex–LMWP interaction term. All scales except conduct problems and leadership included 161 subjects. Conduct problems and leadership items are queried only on the 6- to 11-year BASC-PRS, so the number of subjects with these scales was 149.

**Table 4:** Comparison of betas from metabolite-specific models to the LMWP molar sum for the BASC among boys in a multiethnic birth cohort, Mount Sinai Medical Center, New York, New York, USA, 1998–2008.

BASC domain	LMWP sum	MBP	MEP	MiBP	MMP
Clinical scales					
Aggression	1.24*	1.28*	0.91	−0.12	0.53
Anxiety	0.78	−0.04	0.79	−0.25	1.46
Attention problems	1.29*	0.92	1.28*	0.66	1.66*
Atypicality	0.95	0.83	0.74	0.53	1.13
Conduct problems	2.40*	0.92	1.85*	0.23	1.18
Depression	1.18*	0.78	0.97*	0.29	2.07*
Hyperactivity	1.03	1.34	0.83	0.85	0.61
Somatization	0.36	0.84	0.11	1.04	1.06
Withdrawal	0.46	−0.10	0.44	−0.01	−0.84
Adaptive scales					
Adaptability	−1.08*	−0.92	−0.97*	−1.32*	−1.19
Leadership	−0.88	−0.54	−0.84	−1.30	−1.46
Social skills	−1.04	−0.75	−0.97	−0.93	−0.92
Composite scales					
Externalizing problems	1.75*	1.36*	1.33*	0.33	0.70
Internalizing problems	0.99	0.66	0.80	0.46	1.99*
Adaptive skills	−0.98	−1.18	−0.79	−1.17	−0.85
BSI	1.55*	1.23	1.32*	0.47	1.77*

Abbreviations: MEP, monoethyl phthalate; MiBP, mono-isobutyl phthalate. Includes subjects with *F*-scores of 0–3 and adjusted for race, sex, educational level of the primary caretaker, marital status of the primary caretaker, and urinary creatinine.

* *p* ≤ 0.05.

**Table 5:** Prenatal metabolite concentrations of LMWP and the BRIEF in a multiethnic birth cohort, Mount Sinai Medical Center, New York, New York, USA, 1998–2008.^a^

BRIEF scale/index	β per log_e_-unit increase in LMWP (95% CI)
Inhibit	0.91 (−0.27 to 2.10)
Shift	0.61 (−0.59 to 1.81)
Emotional control	1.33 (0.18 to 2.49)
BRI	1.13 (−0.09 to 2.35)
Initiate	0.81 (−0.27 to 1.89)
Working memory	1.03 (−0.25 to 2.43)
Plan/organize	1.02 (−0.20 to 2.25)
Organization of materials	0.38 (−0.77 to 1.53)
Monitor	0.97 (−0.13 to 2.06)
MI	1.05 (−0.11 to 2.20)
GEC	1.23 (0.09 to 2.36)

a Adjusted for race, sex, educational level of the primary caretaker, marital status of the primary caretaker, and urinary creatinine. All scales except plan/organize included 171 subjects. Plan/organize items are queried only on the 6- to 11-year BASC-PRS, so the number of subjects with these scales was 169.

**Table 6:** Comparison of betas from metabolite-specific models to the LMWP molar sum for the BRIEF in a multiethnic birth cohort, Mount Sinai Medical Center, New York, New York, USA, 1998–2008.

BRIEF scale/index	LMWP sum	MBP	MEP	MiBP	MMP
Inhibit	0.91	0.36	0.73	0.21	1.18
Shift	0.61	0.63	0.41	0.64	1.16
Emotional control	1.33*	0.79	1.10*	0.09	2.17*
BRI	1.13	0.67	0.89	0.30	1.77*
Initiate	0.81	0.77	0.68	0.83	1.56*
Working memory	1.03	1.53*	1.02	1.11	2.04*
Plan/organize	1.02	1.31	0.84	0.76	2.00*
Organization of materials	0.38	1.23	0.14	0.97	0.64
Monitor	0.97	0.45	0.75	0.26	1.20
MI	1.05	1.09	0.89	0.70	1.82*
GEC	1.23*	0.98	1.02	0.56	1.89*

Abbreviations: MEP, monoethyl phthalate; MiBP, mono-isobutyl phthalate. Adjusted for race, sex, educational level of the primary caretaker, marital status of the primary caretaker, and urinary creatinine.

* *p* ≤ 0.05.
